# The Mechanism of the Zika Virus Crossing the Placental Barrier and the Blood-Brain Barrier

**DOI:** 10.3389/fmicb.2020.00214

**Published:** 2020-02-20

**Authors:** Chi-Fen Chiu, Li-Wei Chu, I-Chen Liao, Yogy Simanjuntak, Yi-Ling Lin, Chi-Chang Juan, Yueh-Hsin Ping

**Affiliations:** ^1^Department and Institute of Pharmacology, National Yang-Ming University, Taipei, Taiwan; ^2^Institute of Biomedical Sciences, Academia Sinica, Taipei, Taiwan; ^3^Department and Institute of Physiology, National Yang-Ming University, Taipei, Taiwan; ^4^Institute of Biophotonics, National Yang-Ming University, Taipei, Taiwan

**Keywords:** Zika virus, placental barrier, blood-brain barrier, tight junction, transcytosis, single-virus imaging, ZO-1, Occludin

## Abstract

Zika virus (ZIKV) infection causes severe neurological symptoms in adults and fetal microcephaly and the virus is detected in the brain of microcephaly and meningoencephalitis patient. However, the mechanism of ZIKV crossing the physiological barrier to the central nervous systems (CNS) remains elusive. The placental barrier and the blood brain barrier (BBB) protect the fetus from pathogens and ensure healthy brain development during pregnancy. In this study, we used human placenta trophoblasts cells (JEG-3) and human brain-derived endothelial cells (hCMEC/D3) as *in vitro* models of the physiological barriers. Results showed that ZIKV could infect JEG-3 cells effectively and reduce the amounts of ZO-1 and occludin between adjacent cells by the proteasomal degradation pathway, suggesting that the permeability of the barrier differentially changed in response to ZIKV infection, allowing the virus particle to cross the host barrier. In contrast, ZIKV could infect hCMEC/D3 cells without disrupting the BBB barrier permeability and tight junction protein expression. Although no disruption to the BBB was observed during ZIKV infection, ZIKV particles were released on the basal side of the BBB model and infected underlying cells. In addition, we observed that fluorescence-labeled ZIKV particles could cross the *in vitro* placenta barrier and BBB model by transcytosis and the action of transcytosis could be blocked by either low temperature or pharmacological inhibitors of endocytosis. In summary, the ZIKV uses a cell-type specific paracellular pathway to cross the placenta monolayer barrier by disrupting cellular tight junction. In addition, the ZIKV can also cross both the placenta barrier and the BBB by transcytosis. Our study provided new insights into on the mechanism of the cellular barrier penetration of ZIKV particles.

## Introduction

The Zika virus (ZIKV), first isolated from the rhesus monkey in 1947 in Uganda (Dick et al., 1952), is a re-emerging arthropod-borne RNA virus belonging to the *Flaviviridae* family, which also include the dengue virus (DENV), the West Nile virus (WNV), the Japanese encephalitis viruses (JEV) and the yellow fever virus (YFV). Until 2015, most ZIKV infections were deemed as a mild illness with some common symptoms including headache, fever, arthralgia, rash, myalgia, edema, arthritis, vomiting, and non-purulent conjunctivitis (Petersen et al., 2016). However, based on recent epidemics, ZIKV infection in adult is associated with the Guillain-Barre’ syndrome and encephalitis (Brasil et al., 2016; Dos Santos et al., 2016; Soares et al., 2016). Moreover, vertical transmission of ZIKV from mother to fetus is linked to the elevating incidences of the congenital Zika syndrome on fetuses including microcephaly, congenital malformation, and fetal demise (Cordeiro et al., 2016; Coyne and Lazear, 2016; Hoen et al., 2018). For those infants born with a normal head, congenital ZIKV infection may also cause developed postnatal-onset microcephaly, joint disorders, sensorineural hearing loss, and eye abnormalities (Fitzgerald et al., 2018). These studies revealed a wide-spectrum of effects that congenital ZIKV infection has on fetuses, strongly suggesting the importance of understanding the mechanisms of vertical transmission.

To reach the fetus brain from the infected mother, ZIKV needs to pass two major physiological barriers, the placenta and the blood-brain barrier (BBB). The placenta, a highly specialized organ formed only during pregnancy, supports the growth and development of the fetus and is precisely regulated and coordinated to ensure the maximal efficiency of the exchange of nutrients and waste products between the maternal and fetal circulatory systems (Gude et al., 2004). Its principal function is to supply the fetal brain, with oxygen and nutrients (Burton and Fowden, 2015). Moreover, the placenta barrier serves as an essential physiological barrier that protects the fetus from certain toxic molecules, maternal diseases, and pathogenic infections, such as viruses (Gude et al., 2004; Delorme-Axford et al., 2014). The main functional unit of the placenta is the chorionic villi that is composed of specialized epithelial cells known as trophoblasts derived from the outer trophectoderm layer. Trophoblasts construct the epithelial covering of the placenta and also generate a subpopulation of invasive extravillous trophoblast cells within which fetal blood is separated by the placental membrane from the maternal blood (Gude et al., 2004; Burton and Fowden, 2015). To serve as the initial line of defense against any pathogens attempting to breach the placental barrier, trophoblasts constitute a tight polarized epithelial monolayer comprising tight junctions preventing lateral and paracellular diffusion of substrates. However, at the present time, our knowledge regarding virus-host interactions at the maternal-fetal interface during pregnancy is limited.

While the placenta serves as the first checkpoint to protect the fetus and support its normal growth and development of the fetus, the BBB provides the second checkpoint critical to protect the fetal brain and ensure healthy brain development (Coyne and Lazear, 2016). The BBB is a boundary that separates the circulating blood from the brain and the extracellular fluid in the central nervous system (CNS) (Daneman and Prat, 2015). The BBB is made up of endothelial cells of the vasculature forming cell-to-cell tight junctions to limit the passage of circulating molecules, cells, and pathogens to the CNS (Stamatovic et al., 2008). Tight junctions containing more than 40 proteins with both transmembrane and cytoplasmic domains generate a continuous, circumferential, belt-like structure at the luminal end of the intercellular space, where it serves as a gatekeeper of the paracellular pathway (Mateo et al., 2015). Three major transmembrane proteins, claudin, occluding, and junctional adhesion molecule (JAM), interact with cytoplasmic proteins including ZO-1, cingulin, afadin and α-catenin, which anchor strands to the cytoskeleton, resulting in the formation of cellular tight junctions (Hartsock and Nelson, 2008). Disruption of the BBB enhances permeability of endothelial cell and is a hallmark of CNS infection (Daniels and Klein, 2015). However, recent studies reported that no barrier disruption was observed when ZIKV gained access to the CNS (Papa et al., 2017; Alimonti et al., 2018), suggesting that the endocytic transport system is required for the ZIKV to cross the BBB barrier. Given the existence of endosomal sorting pathways in different cell types, it is possible for the BBB cells to employ a similar process of endosomal transportation (Ayloo and Gu, 2019). However, there is no investigation on these pathways in BBB endothelial cells, particularly in the condition under viral infection.

The placental barrier and the BBB protect the fetal brain development during human pregnancy by forming cellular tight junctions to limit pathogen paracellular movement under the normal condition. However, the ZIKV could be detected in the brains of microcephalic infants, suggesting that the ZIKV can penetrate the CNS in fetus (Calvet et al., 2016; Mlakar et al., 2016). There are two possible routes for the viruses to cross the physiological barriers. One is to disrupt the barrier integrity. The Rubella virus, cytomegalovirus (CMV), the Human immunodeficiency virus type 1 (HIV-1), the West Nile virus (WNV), the Japanese encephalitis virus (JEV), and herpes viruses are known to breach the placental barriers to reach the CNS (Roe et al., 2012, 2014; Coyne and Lazear, 2016; Al-Obaidi et al., 2017; Leibrand et al., 2017; Mittal et al., 2017). The other is through transcytosis. The hepatitis B virus (HBV) has been reported to penetrate the placenta barrier by transcytosis in the first trimester (Bhat and Anderson, 2007). The JEV has also been found to cross the endothelial cells and pericytes in the BBB in endocytic vesicles (Liou and Hsu, 1998). These findings indicate that viruses may cross the barrier not only through the paracellular pathway but also through transcytosis. Although the ZIKV is detected in both the amniotic fluid of pregnant women and in microcephalic fetal brain tissues (Calvet et al., 2016; Mlakar et al., 2016), and is thus capable of penetrating the CNS from mother to fetus, the mechanism of such remains unknown. In this study, we would like to combine the *in vitro* Transwell barrier assay and a single-virus tracking (SVT) approach to elucidate the mechanism that the ZIKV employs to cross the placental and the BBB barriers.

## Materials and Methods

### Cell Culture

The choriocarcinoma cell lines (JEG-3, ATCC^®^ HTB-36^TM^) and African green monkey kidney epithelial cells (Vero, ATCC^®^ CCL-81^TM^) were cultured in a minimum essential medium (MEM, Gibco) supplemented with 10% fetal bovine serum (FBS, HyClone), 1% L-glutamine and 1% penicillin/streptomycin (P/S, Gibco). The immortalized human brain capillary endothelial cell line hCMEC/D3 (SCC066, Merck) was cultured in the EndoGRO^TM^-MV Complete Media Kit (Merck). The Vero E6 cell line was cultured in Dulbecco’s modified Eagle’s medium (DMEM, Gibco) supplemented with 10% fetal bovine serum and 1% penicillin/streptomycin. All cells were incubated at 37°C with 5% CO_2_.

### ZIKV Amplification

The ZIKV strain PRVABC59 (2015 Puerto Rico strain, GenBank accession: KU501215) kindly provided by the Centers for Disease Control, Taiwan, was propagated in Vero E6 cells. Cells were exposed to ZIKV with multiplicity of infection (MOI) of 0.02 in a serum free DMEM medium and were incubated at 37°C with 5% CO_2_ for 2 h. Afterward, infected cells were replaced in a low serum DMEM medium containing 2% FBS, 1% P/S for virus production. At 4th and 7th days post-infection, the culture medium was collected and cell debris was removed by centrifugation at 1500 × *g* for 15 min at 4°C. Finally, the ZIKV-containing supernatant was stored at −80°C.

### Immunofluorescence Microscopy Imaging

For ZIKV infection assays, JEG-3 and hCMEC/D3 cells were seeded on 3.5 cm dishes and incubated overnight until cells grown to 80% confluent monolayer, and cells were then infected with different MOIs of the ZIKV. After 24 h post-infection, cells were fixed by 4% paraformaldehyde, and permeabilized by 0.1% Triton X-100 in PBS. The expression of the ZIKV E protein was recognized by a 1:100 diluted rabbit anti-ZIKV E antibody (GeneTex, GTX133314) and Alexa-488 conjugated goat anti-rabbit IgG. Nuclear DNA was stained with 4,6-diamidino-2-phenylindole, dihydrochloride (DAPI; Sigma-Aldrich). Fluorescence images were captured by an Olympus IX70 microscope equipped with a 20 × objective lens. The percentage of ZIKV-infected cells was calculated by (ZIKV E positive cells/total cells) × 100%. The average percentages in ZIKV-infected cells were collected from three independent experiments. For tight junction protein expression assays, JEG-3 cells were seeded on a 3.5 cm glass-bottom plate (Mettek) until cells grown to 100% confluent monolayer, and then incubated with/without the ZIKV. After 24 h post-infection, the cells were fixed by 4% paraformaldehyde and incubated with ZO-1 (Invitrogen) or occludin (Abcam) antibodies. Afterward, cells were recognized by the Alexa-488 conjugated secondary antibody, and nuclear DNA was stained with DAPI (Sigma-Aldrich). Fluorescence images were captured by a FluoView 1000 confocal microscope (Olympus) equipped with a 60 × oil immersion objective with a numerical aperture (N.A.) of 1.4.

### Cell Viability Assays

To determine cell viability in the presence of various inhibitors or drugs, a sulforhodamine beta (SRB) assay was performed. JEG-3 and hCMEC/D3 cells were seeded on 96-well plates. After a 24-h treatment, cells were fixed with 10% trichloroacetic acid at 4°C for 1 h. Cells were washed with water prior to incubation of 100 μl of 0.5% sulforhodamine beta in 1% acetic acid for 30 min at RT. Plates were washed four times with 1% acetic acid and air-dried. SRB was dissolved in 50 μl of 10 mM Tris solution and absorbance was measured at 510 nm using an ELISA reader.

### *In vitro* Transwell Barrier Assay

To assess the ability of the ZIKV crossing the barrier *in vitro*, the virus was added to the media in the apical Transwell insert (translucent polyethylene terephthalate [PET], 0.4 μm pore size). The inoculum was left on the cells for 24 h except for the experiment in which the inoculum was removed after 2 h and replaced with complete endothelial medium for additional 24 h. The inserts were placed in on a 24-well companion plate containing Vero cells growing at the bottom of the well. After 24 h post-infection, Vero cells were washed with PBS, and recognized by an anti-ZIKV E protein antibody and ALEXA488 conjugated goat anti-rabbit IgG, sequentially. Nuclear DNA was stained with DAPI (Sigma-Aldrich). The percentage of ZIKV-infected cells was calculated by (ZIKV E positive cells/total cells) × 100%.

### *In vitro* Transwell Permeability Assays

To test whether the permeability of the physiological barrier was affected by the ZIKV, FITC-dextran was added to the media in the apical Transwell insert after the barrier cells were exposed to the ZIKV for 24 h. The cells were seeded on the membrane insert in the Transwell chamber, as previously described. Before FITC-dextran was added to the media in the apical insert, the inserts were washed twice with a pre-warmed Krebs-HEPES buffer (99 mM NaCl, 4.7 mM KCl, 1.2 mM MgSO_4_, 1.0 mM KH_2_PO_4_, 19.6 mM NaHCO_3_, 11.2 mM glucose, 20 mM Na -HEPES, and 2.5 mM CaCl_2_, pH 7.4) to remove the residual medium. The inserts were then placed onto companion plates containing 500 μl of Krebs-HEPES buffer. Afterward, dextran conjugated with FITC (FITC-dextran 1 mg/ml) was added to the apical insert and incubated at 37°C for 1 h. The insert was removed and the solution in the plate well (lower chamber) was collected and measured for fluorescence intensity of FITC-dextran using a fluorescent plate reader (excitation 492 nm and excitation 518 nm).

### Western Blotting

Infected cells were lysed in RIPA buffer (20 mM Tris–HCl pH 7.5, 150 mM NaCl, 1 mM Na_2_EDTA, 1 mM EGTA, 1% NP-40, 1% sodium deoxycholate, 2.5 mM sodium pyrophosphate, 1 mM beta-glycerophosphate, 1 mM Na3VO4, 1 μg/ml leupeptin, and 1 mM PMSF). Proteins were separated by 10% SDS-PAGE and transferred to polyvinylidene fluoride membranes which were blocked with a blocking buffer (5% skim milk in TBS with 0.05% Tween 20) and incubated with primary antibodies in the blocking buffer. Herein, the rabbit polyclonal anti-ZO-1 (Invitrogen), the rabbit polyclonal anti-Zika virus E protein (GeneTex, GTX133314), the rabbit monoclonal anti-Occludin antibody (Abcam), and the rabbit polyclonal anti- GAPDH were utilized, respectively. After being washed three times with a blocking buffer, the membrane was probed with a horseradish peroxidase-conjugated secondary antibody and developed with an Immobilon chemiluminescent HRP substrate (Millipore). Blots were imaged on an Amersham Imager 680.

### RT-qPCR Analysis

Total RNA was extracted using a TRIzol reagent (Invitrogen, Thermo Scientific-Technologies). cDNA was synthetized from total RNA (5 μg) using RevertAidTM reverse transcriptase (Thermo Fisher Scientific). Real-time PCR amplification was performed using the KAPA SYBRTM FAST qPCR Kits on BiosystemsTM Real-Time PCR Instruments. Specific primers for individual genes used for RT-qPCR are: ZO-1 (Forward: 5′- CACCTTTTGATAATCAGCACTCTCA-3′; Reverse: 5′- CTCTAGGTGCCTGTTCGTAACGT-3′), Occludin (Forward: 5′- TCAGGGAATATCCACCTATCACTTCAG-3′; Reverse: 5′- CATCAGCAGCAGCCATGTACTCTTCAC-3′), ZIKV E protein (Forward: 5′- AAGTACACATACCAAAACAAAGTGGT-3′; Reverse: 5′- TCCGCTCCCCCTTTGGTCTTG-3′), and GAPDH (Forward: 5′- AAGGTCATCCCTGAGCTGAA-3′; Reverse: 5′-TTCTAGACGGCAGGTCAGGT-3′). Amplification of cDNA was initiated with 3 min at 95°C, followed by 50 cycles of 3 s at 95°C and 30 s at 60°C.

### Preparation of Fluorescence-Labeled ZIKV Particles

The procedure of virus labeling was performed as previously described with minor modifications (Chu et al., 2014, 2019). ZIKV particles were pelleted from a ZIKV-containing supernatant by ultra-centrifugation at 47,000 rpm in a Beckman 50.2Ti rotor for 3.5 h. Virus pellets were resuspended in a HNE buffer (5 mM HEPES, 150 mM NaCl, and 0.1 mM EDTA, pH 7.4) and further concentrated by ultrafiltration spin columns (GE healthcare). Concentrated ZIKV particles were labeled with Atto647N-NHS ester (Sigma-Aldrich), with maximum absorption at 646 nm and maximum emission at 664 nm. Briefly, 1 × 10^7^pfu/ml of ZIKV were mixed with 2 nmol of Atto 647N NHS ester in an HNE buffer for 45 min at room temperature. The unincorporated dye was separated from fluorescence-labeled ZIKV particles by a Sephadex G-25 column (GE Healthcare). The fractions containing Atto647N-labeling ZIKV were detected by a multimode microplate reader (TECAN 200/200Pro) and stored at −80°C.

### Plaque Assay

Vero E6 cells were seeded in a 12-well plate with a density of 1.5 × 10^5^ and incubated at 37°C with 5% CO_2_ overnight. After the cells reached 80–90% confluent, they were washed with PBS and were infected with 10-fold serial dilutions of the ZIKV at 37°C with 5% CO_2_ for 2 h. Afterward, ZIKV-infected cells were overlaid with a 1:1 mixture of 2% agarose gel and two-fold DMEM medium containing 4% FBS and 1% P/S, and then incubated at 37°C with 5% CO_2_. At 4 days post-infection, the cells were fixed by 10% formaldehyde, and the gels were removed. Finally, the cells were stained with 1% crystal violet to visualize plaque formation. The virus titer was quantified by counting the plaque numbers.

### *In vitro* Barrier Transcytosis Assays

Cells were seeded to 24-well PET cell culture inserts, 0.4-μm pore size for 7 days, in a two-chambered system. In this system, barrier cells form a polarized monolayer with tight junctions between cells, allowing access to both apical and basolateral domains. Cells were incubated for 30 min with various transcytosis inhibitors in different concentration, including 10 μM of nystatin (Sigma), 10 μM of chlorpromazine hydrochloride (CPZ, Sigma), 20 μM of dimethyl amiloride (DMA, Sigma), and 10 μM of colchicine (Sigma). Drugs were administered to the cells in both apical and basolateral chambers at identical concentrations. Thereafter, 100 μl of Atto647-ZIKV (2 × 10^6^ copies ml^–1^ of virus) was added into the upper chamber. After 4 h incubation with cells, all of the basolateral supernatant was collected. Percent transcytosis was determined by measuring the fluorescence intensity of Atto647N through using a fluorescent plate reader.

### Statistics Analysis

The student *t*-test was used for statistical analyses in this study. The repeat times for each experiment are described in the figure legends or main text. The software Prism 6 (GraphPad) was used to perform statistical analysis. Statistical significance is defined as, n.s., not significant, ^∗^*p* < 0.05, ^∗∗^*p* < 0.01, ^∗∗∗^*p* < 0.001, ^****^p < 0.0001.

## Results

### ZIKV Infects Both JEG-3 Cells and hCMEC/D3 Cells With No Effect on Cell Viability

**FIGURE 1:**
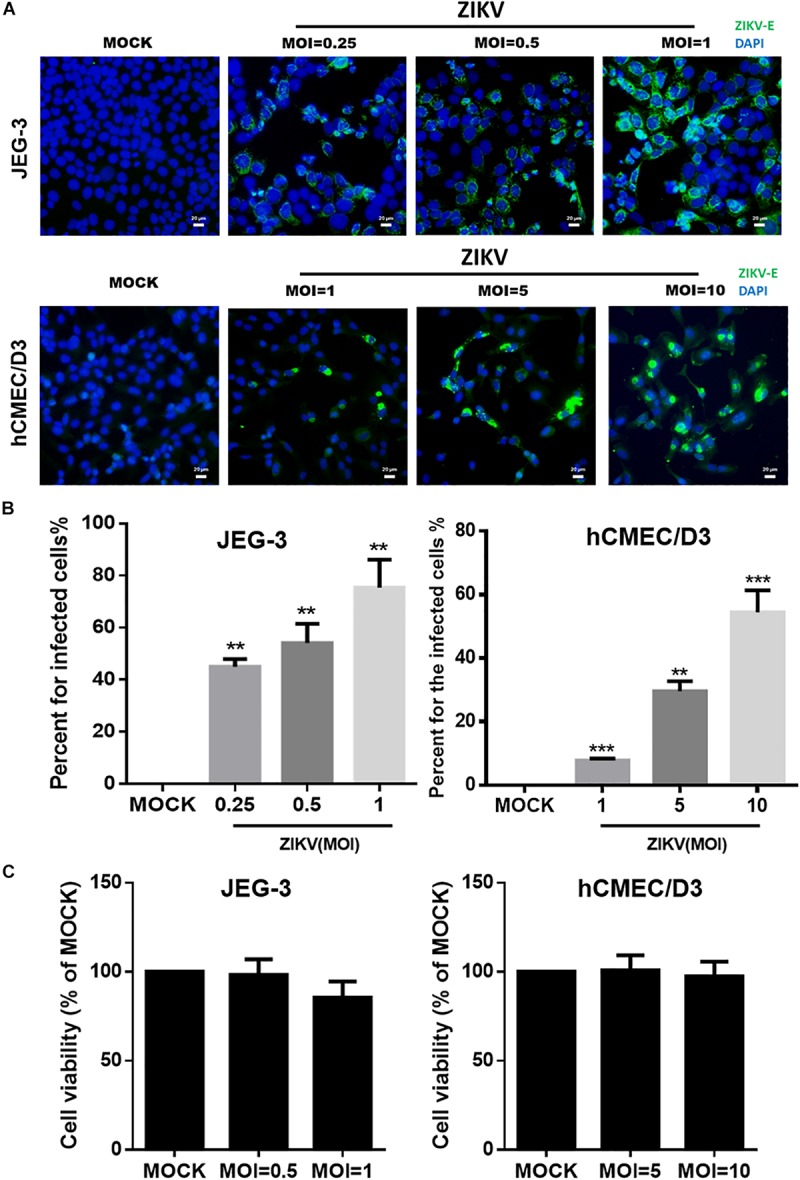
The infectivity and virulence of ZIKV in JEG-3 and hCMEC/D3 cells. **(A)** The immunofluorescence images of ZIKV E protein revealed the population of ZIKV-infected cells in JEG-3 (MOI of 0.25 to 1) and hCMEC/D3 cells (MOI of 1 to 10) at 24 h post-infection. The ZIKV E protein antibody was recognized by an Alex488-secondary antibody (green). Nuclear DNA was stained with DAPI (blue). Scale bar, 20 μm. **(B)** The percentage of infected cells was quantified from **(A)**. **(C)** JEG-3 cells were infected ZIKV at MOI of 0.5 to 1, and hCMEC/D3 cells were infected ZIKV at MOI of 5 to 10 during 24 h. The virulence of ZIKV in JEG-3 and hCMEC/D3 was measured by a cell viability assay. The error bars represent standard deviations from three independent experiments. Statistical differences were obtained through *t*-tests. **p < 0.01; ***p < 0.001.

To investigate the mechanism of the ZIKV crossing the placental barrier and the BBB, we infected JEG-3 cells, which are derived from trophoblast cells of the placenta and commonly used in plancental barrier studies, and hCMEC/D3 cells, which are the immortalized human brain capillary endothelial cell lines frequently employed in studies of the BBB (Vu et al., 2009; Rothbauer et al., 2017). To first clarify the infectivity of the ZIKV in JEG-3 and hCMEC/D3 cells, the viral envelop (E) protein was detected by immunofluorescence staining with an anti-ZIKV E protein antibody at 24 h post-infection. Apparently, the ZIKV E protein was detected in both cell lines in a dosage-dependent manner (Figure 1A). Results of the quantitative analysis revealed that the percentage of infected cells could be elevated as MOI increased (Figure 1B). The ZIKV infected more than 80% cells at a MOI of 1 and 10 in JEG-3 and hCMEC/D3 cells, respectively (Figure 1B). Importantly, there was no cytotoxicity in cell viability of both cell lines among indicated MOI at 24 h post-infection (Figure 1C).

### ZIKV Crosses an *In vitro* Human Physiological Barrier Model

**FIGURE 2:**
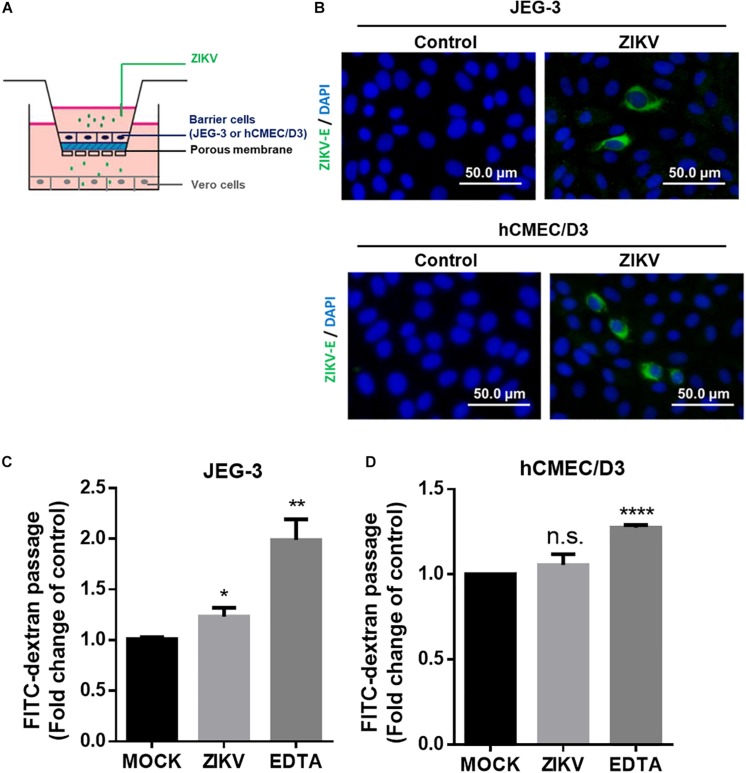
ZIKV crossed *in vitro* human physiological barriers. **(A)** A schematic diagram depicts a virus crossing *in vitro* barrier cells. **(B)** The immunofluorescence images of the ZIKV E protein revealed ZIKV-infected Vero cells cultured in a basal chamber through a JEG-3 barrier at a MOI of 0.5 or hCMEC/D3 barrier at MOI of 10 at 24 h post-infection. The ZIKV E protein antibody was recognized by an Alex488-secondary antibody (green). Nuclear DNA was stained with DAPI (blue). Scale bar, 50 μm. **(C)** FITC-dextran (40 kDa) was used to validate the permeability of a JEG-3 barrier at 24 h post-infection. The FITC-dextran of the basal chamber was collected and analyzed by a plate reader. The fold change of the FITC signal compared with the mock group was drawn in **(C)**. 12.5 μM of EDTA was used as a positive control. The permeability of an hCMEC/D3 barrier at 24 h ZIKV post-infection was measured in **(D)**. The error bars represent standard deviations from three independent experiments. *p < 0.05; **p < 0.01; ****p < 0.0001.

Next, we established an *in vitro* Transwell barrier assay to investigate whether the ZIKV can cross barrier cells. As shown in Figure 2A, JEG-3 and hCMEC/D3 cells were, respectively, seeded on the apical chamber of the inserts, which contained a permeable membrane, of a 24-well Transwell plate. The inserts were then placed on a 24-well companion plate containing pre-seed adherent Vero cells in the basal chamber. ZIKV was added on the top of the inserts at a MOI of 0.5. If ZIKV particles could cross barrier cells, they would exist in the basal chamber and infect into Vero cells. The existence of the ZIKV in Vero cells was detected by immunostaining using an anti-ZIKV E protein antibody. The results showed clearly that the ZIKV E protein could be detected in Vero cells in either JEG-3 and hCMEC/D3 cells as barrier cells (Figure 2B). These results indicated that the ZIKV could cross the human physiological barrier cells. It is possible for the ZIKV to cross barrier cells by altering the permeability of barrier cells. To investigate this possibility, the permeability of the cells was examined by using FITC-dextran as an indicator in the *in vitro* Transwell barrier assay. FITC-dextran was added in the apical chamber after 24 h post-infection. If ZIKV infection could alter cell permeability, the signal of FITC-dextran should be detected in the basal chamber. Compared with the mock group, ZIKV infection did increase FITC-dextran signal significantly when JEG-3 cells were used as barrier cells (Figure 2C). In contrast, there was no significant change of the FITC-dextran signal between the mock group and the ZIKV-infected group when hCMEC/D3 cells were used as barrier cells (Figure 2D). Ethylenediaminetetraacetic acid disodium salt (EDTA) that can increase cell permeability was used as a positive control. These results suggested that the ZIKV may cross the placenta barrier by changing the permeability of the barrier cells. In contrast, the ZIKV crosses the BBB barrier cells but does not change the permeability of the BBB barrier cells (Figures 2B,C), suggesting no barrier leakage when the ZIKV crossed the BBB.

### ZIKV Down-Regulated the Expression of Tight Junction Protein Through a Proteasomal Degradation Pathway

**FIGURE 3:**
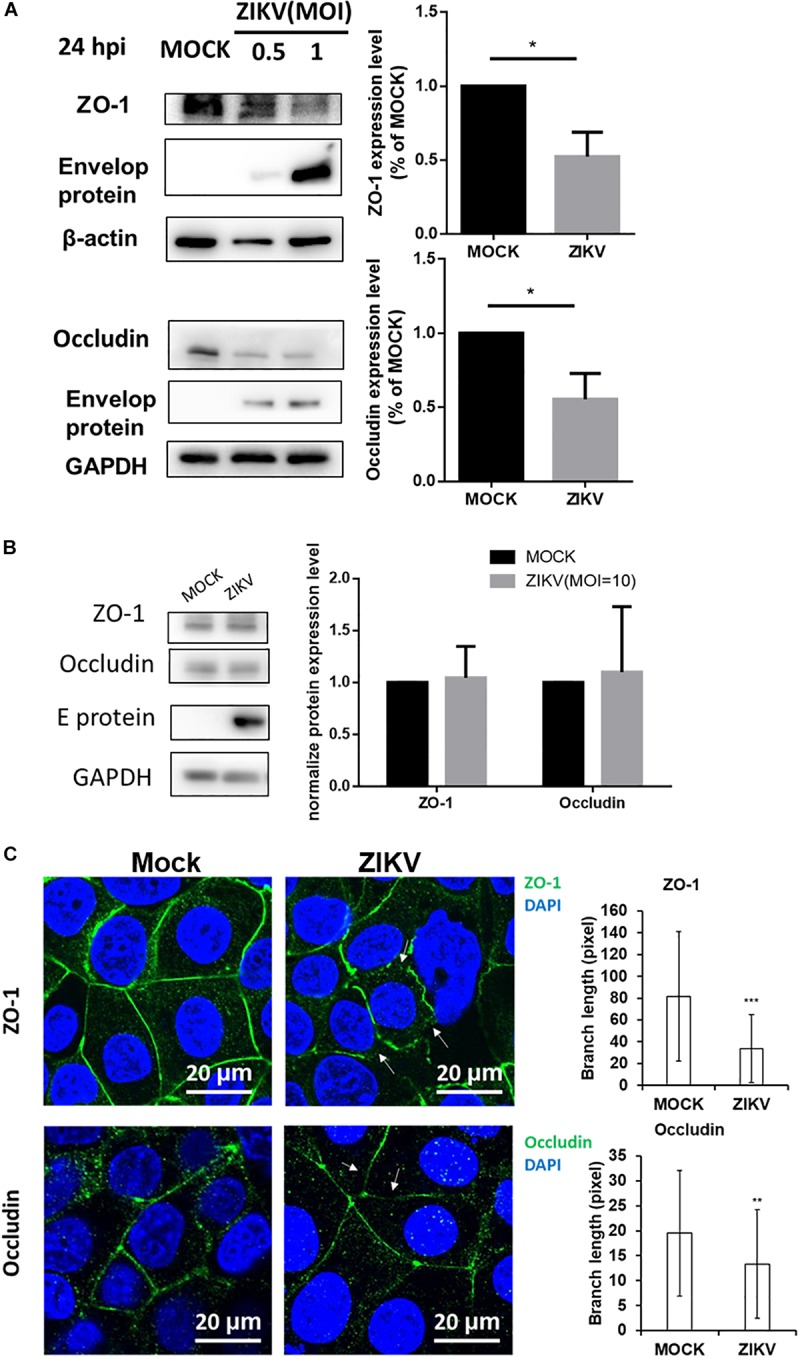
ZIKV infection decreased expression of the tight junction protein ZO-1 and Occludin in JEG-3 but not hCMEC/D3. **(A)** Western blotting depicted that the amount of ZO-1 and Occludin decreased in JEG-3 cells at 24 h post-infection. The amount of ZO-1 and Occludin was normalized by β-actin and GAPDH, respectively. Comparison with the mock group was quantified in the right panel. Statistical differences were obtained through *t*-tests. **p < 0.05. **(B)** Western blotting depicted that the expression of ZO-1 and Occludin were not affected in hCMEC/D3 cells with ZIKV infection. The amount of ZO-1 and Occludin was normalized by GAPDH, and quantified in the right panel. **(C)** The distribution of ZO-1 and Occludin in JEG-3 cells with/without ZIKV infection were imaged by confocal microscopy. The white arrows indicate the disruption of ZO-1 and Occludin. Scale bar, 20 μm. The tight junction integrity of ZO-1 and occludin were measured by the Skeletonize plug-in of ImageJ software. The length of segments in each group (*n* = 50) were measured and quantified as right histograms. **p < 0.01, ***p < 0.001.

Given that the leakage of FITC-dextran was increased by ZIKV infection in the *in vitro* placenta barrier model but not in the BBB barrier model (Figures 2C,D), it raised a possibility that the disruption of the tight junction of JEG-3 is associated with the barrier leakage because the tight junctions of trophoblasts epithelial cells bear critical barrier functions for protection against a paracellular spread of various pathogens, including viruses (Delorme-Axford et al., 2014). To investigate this hypothesis, the expression of two tight junction proteins, ZO-1 and occludin were examined in JEG-3 as well as hCMEC/D3. The results of the western blotting assay clearly depicted a decreasing expression of ZO-1 and occludin in JEG-3 cells in the presence of ZIKV infection compared to that in the mock control group (Figure 3A). In contrast, there was no significantly different expression of ZO-1 and occludin between the ZIKV-infected group and the mock control group in hCMEC/D3 cells (Figure 3B). To further confirm the disruption of tight junction by ZIKV infection in JEG-3 cells, the distribution of ZO-1 and occludin were detected by an immunofluorescence assay. Confocal imaging revealed that tight junctions formed continuous seals between adjacent cells in the absence of ZIKV infection (Figure 3C). In contrast, ZIKV infection disrupted the continuity of occludin and ZO-1 in JEG-3 cells (white arrows in Figure 3C, left panel). The integrity of junctional ZO-1and occludin of JEG-3 cells was quantified by measuring the branch length of segments. The results demonstrated that ZIKV infection declined the integrity of tight junction of JEG-3 cells (Figure 3C, right panel).

**FIGURE 4:**
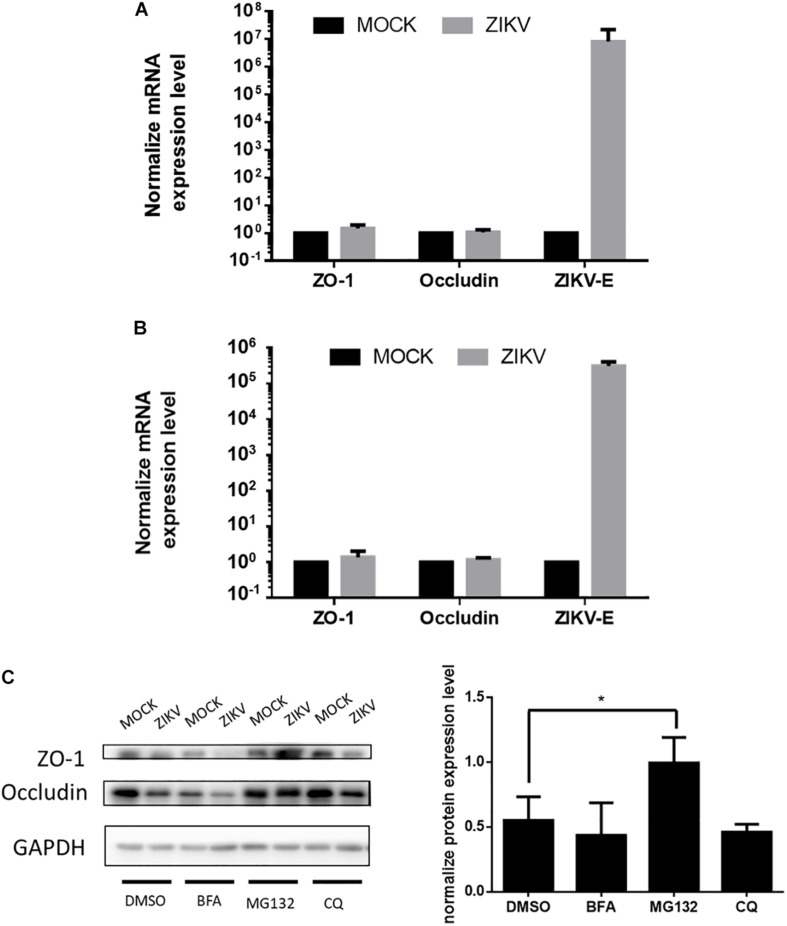
ZIKV infection decreased the accumulation of tight junction proteins through post-transcription regulation. **(A)** The mRNA levels of ZO-1, Occludin and ZIKV-E in JEG-3 cells upon ZIKV infection were quantified by RT-qPCR. Data were normalized by GAPDH. The fold changes of mRNA were compared with the mock group. The mRNA levels of ZO-1, Occludin and ZIKV-E in hCMEC/D3 upon ZIKV infection are shown in **(B)**. The error bars represent standard deviations from three independent experiments. **(C)** Western blotting depicts the accumulation of ZO-1 and Occludin in JEG-3 cells with various inhibitors treatments upon ZIKV infection. The amount of ZO-1 and Occludin was normalized by GAPDH. The fold changes of protein expression compared with the mock group in each term were quantified in the right panel. The error bars represent standard deviations from three independent experiments. *p < 0.05.

To further investigate the regulatory mechanism of ZIKV-induced decreased of ZO-1 and occludin expression, mRNA levels of these two proteins isolated from JEG-3 and hCMEC/D3 cells in the absence or presence of ZIKV infection were determined by RT-qPCR. The results of RT-qPCR depicted no significant change of mRNA level of ZO-1 and occludin in both JEG-3 and hCMEC/D3 cells regardless of ZIKV infection (Figures 4A,B). These data suggested that the down regulation of ZO-1 and occludin expression in JEG-3 cells might be regulated at the post-transcription level. To confirm this speculation, prior to ZIKV infection, JEG-3 cells were treated individually by a series of pharmacological inhibitors including bafilomycin (BFA), MG132, and chloroquine (CQ), which target either autophagic or proteasomal degradation pathways, the two major cellular pathways mediating protein degradation. As shown in Figure 4C, the expression of ZO-1 and occludin repressed by ZIKV infection could be notably rescued only by MG132, a proteasome inhibitor, but not by either BFA, an inhibitor for both V-ATPase-dependent acidification and autophagosome-lysosome fusion or CQ, an inhibitor for fusion of autophagosomes and lysosomes. Taken together, these results demonstrated that ZIKV infection causes the leakage of the placenta barrier through the disruption of cellular tight junction via the proteasomal degradation pathway.

### Visualization of Transcytosis of ZIKV

**FIGURE 5:**
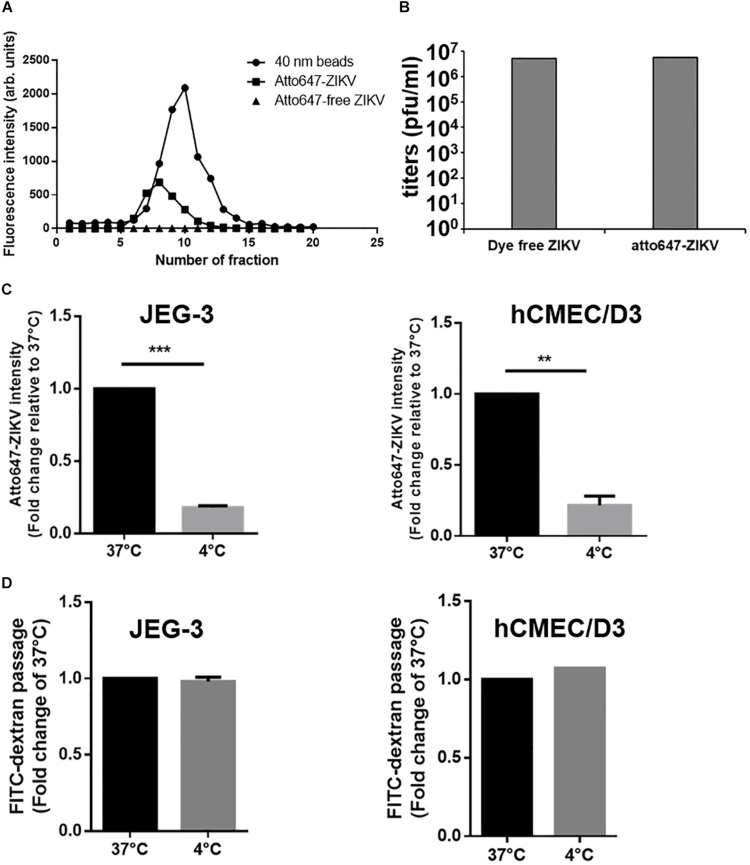
Detection of ZIKV transcytosis. **(A)** Fluorescence intensity profiles of elution from a Sephadex G-25 size-exclusion column is shown. An Atto647N-ZIKV particle was purified within the fraction layer 6 to 10 from gel filtration (solid squares). No fluorescence signal was detected in Atto647N-free ZIKV (solid triangles) within the same fraction layers. The 40 nm fluorescence microspheres were used as a size marker (solid circles). **(B)** Plaque assays were performed to determine the infectivity of the Atto647N-ZIKV. Dye-free ZIKV was used as a control. Both virus samples were purified from the same fractions of the Sephadex G-25 column. **(C)** Atto647N-ZIKV was used to validate virus crossing efficiency in JEG-3 and hCMEC/D3 barriers at 37 or 4 °C. **(D)** FITC-dextran was used to validate the permeability of JEG-3 and hCMEC/D3 barriers in **(C)** condition. The error bars represent standard deviations from three independent experiments. **p < 0.01, ***p < 0.001.

It has been reported that transcytosis, a series action to transport macromolecules intracellularly through the vesicular system, is a common strategy for molecules crossing impermeable barriers under normal physiological condition (Tuma and Hubbard, 2003). Therefore, we would like to conduct a single-virus imaging analysis to investigate whether ZIKV particle can also cross barriers cells by transcytosis in *in vitro* Transwell barrier models. To visualize single ZIKV particles directly, ZIKV particles were labeled with a fluorescence dye, atto647N, by conjugating atto647N-NHS ester with the amino group of the viral envelope protein. The atto647N-labeled ZIKV particles were purified through a Sephadex G-25 size-exclusion column. Compared to the fractions containing the 40 nm fluorosphere (Figure 5A, solid circles), the fluorescent signals of atto647N existent from Fraction 6 to Fraction 10 (Figure 5A, solid squares) indicated atto647N-labeled ZIKV particles (atto647N-ZIKV) because the diameter of a single ZIKV particle is approximately 50 nm (Sirohi et al., 2016; Sevvana et al., 2018). No fluorescence signal was detected within the same range of fractions from the Dye-free ZIKV group (Figure 5A, solid triangles). These results confirmed a successful conjugation of ZIKV particles with atto647N. The infectivity of atto647N-ZIKV particles was then measured by a plaque assay. Both dye-free ZIKV and atto647N-ZIKV presented similar virus titers, suggesting no significant influence on ZIKV infectivity by the atto647N labeling procedure (Figure 5B).

**FIGURE 6:**
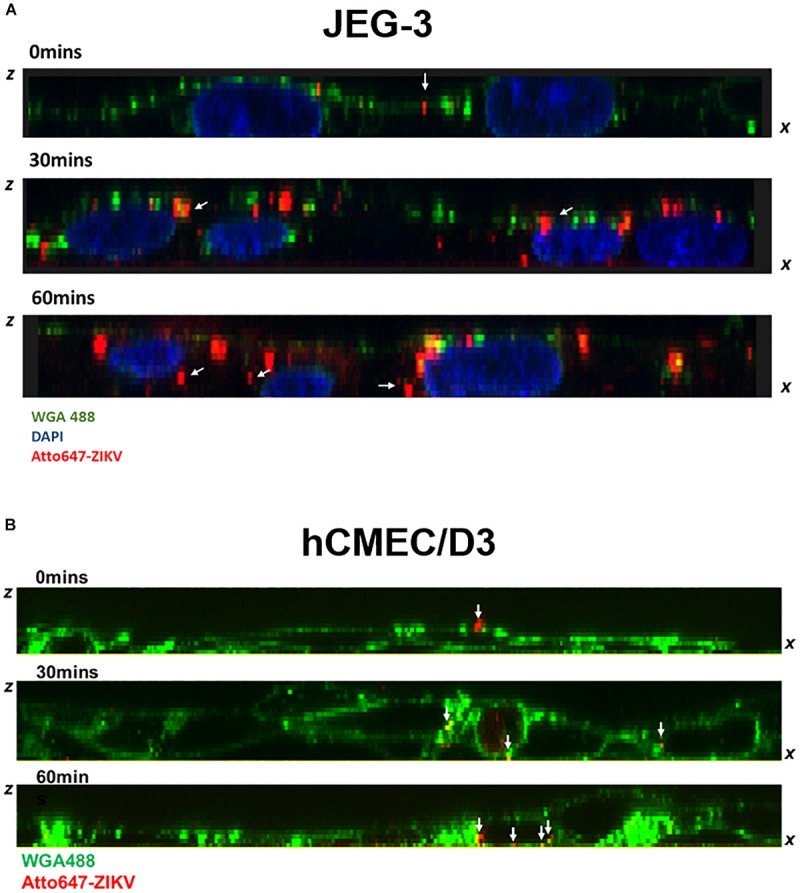
Visualization of ZIKV transcytosis. The ZX images of confocal microscopy depicted the distribution of atto647-ZIKV from the apical side to the basolateral side within JEG-3 cells **(A)** and hCMEC/D3 cells **(B)** at 0, 30, and 60 min post-infection. The white arrows indicated att647-ZIKV particles (red) **(A,B)**. Nucleus was stained by DAPI staining (blue in **A**), and cell membrane was stained by WGA488 (green) **(A,B)**.

Given that transcytosis is a characteristic pathway of intracellular trafficking that allows a selective and rapid transcellular transport from the apical side to the basolateral side within a cell, and the intracellular transport is a temperature-dependent process that can be inhibited at 4°C, in contrast to the paracellular transport (Morad et al., 2019), we speculated that the energy-dependent intracellular transport of virus particles should be inhibited at 4°C. Therefore, using an *in vitro* Transwell barrier assay, we would like to investigate whether the ZIKV can penetrate a monolayer of barrier cells at 4°C and 37°C by directly measuring the fluorescence intensity of atto647N signals in the basal chamber of a Transwell plate. In both JEG-3 and hCMEC/D3 cells, atto647N-ZIKV particles were significantly decreased in the basal chamber at 4°C condition, compared to that at 37°C (Figure 5C). In contrast to atto647N-ZIKV particles, there was no difference of FITC-dextran in the basal chamber of Transwell plate at both 4°C and 37°C (Figure 5D). These results implied that the ZIKV can cross monolayers of either JEG-3 or hCMEC/D3 cells through a temperature-dependent transcytosis. To directly visualize the transcytosis of ZIKV particle across a monolayer of barrier cells, we infected a monolayer of JEG-3 cells on a 3.5 cm glass-bottom plate with Atto647N-ZIKV and acquired confocal imaging at 0, 30, and 60 min post-infection. JEG-3 cells were stained with DAPI and WGA488 to indicate the positions of nucleus and cell membrane, respectively. As shown in Figure 6A, Atto647N-ZIKV particles were only detected on the surface of the JEG-3 monolayer at the initial time point (0 min). Apparently, Atto647N-ZIKV particles were internalized into cytoplasm and moved from the apical side to the basal side of the cells at 30- and 60-min post-infection (Figure 6A). The similar results also can be observed in hCMEC/D3 monolayers (Figure 6B). These results elucidated that ZIKV particles might be directly transported across the monolayer of barrier cells by transcytosis without undergoing the viral replication process.

### ZIKV Transport Can Be Blocked by Endocytosis and Microtubule Inhibitors

**FIGURE 7:**
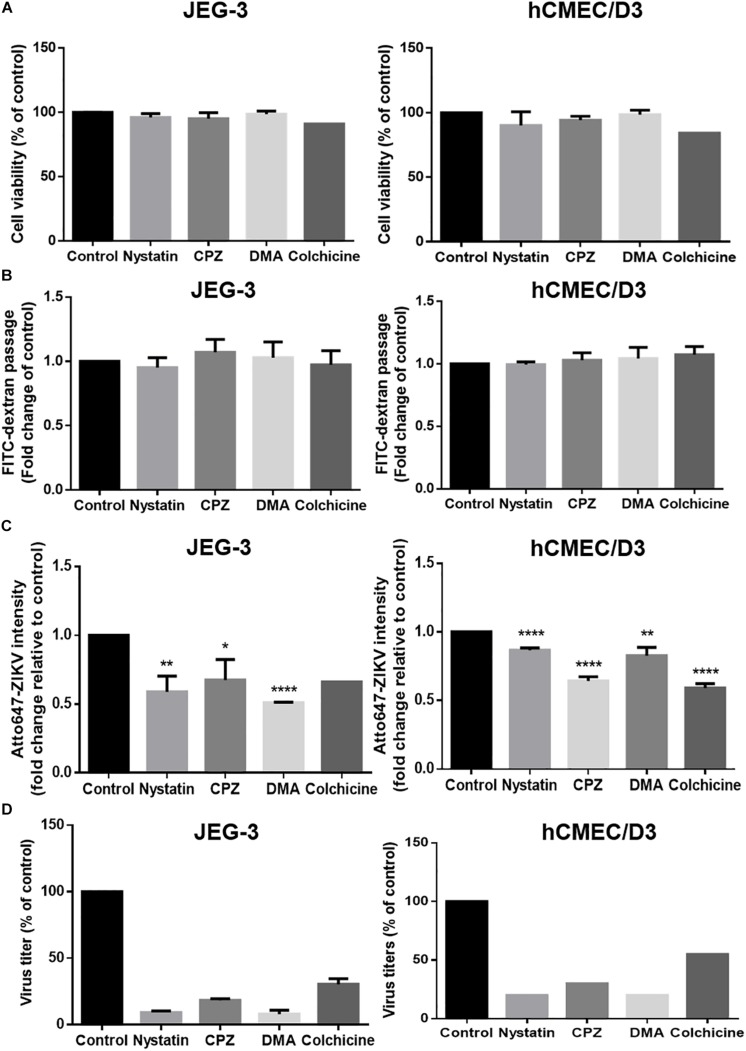
ZIKV transcytosis across JEG-3 and hCMEC/D3 barriers can be reduced by endocytosis and microtubule inhibitors. **(A)** The cell viabilities of JEG-3 and hCMEC/D3 with/without various inhibitors treatment were analyzed by a SRB assay. **(B)** The permeability of JEG-3 and hCMEC/D3 barriers with/without various inhibitors treatment were validated by detecting FITC-dextran across cell barriers. **(C)** Atto647-ZIKV was used to validate virus crossing efficiency in JEG-3 and hCMEC/D3 barriers with/without inhibitors treatment. **(D)** A plaque assay was used to measure the virus titers across JEG-3 and hCMEC/D3 barriers with/without various inhibitor treatments. All results are depicted as fold changes compared to the non-treatment control. The error bars represent standard deviations from three independent experiments. *p < 0.05; **p < 0.01; ****p < 0.0001.

Endocytosis recognized as the responsible mechanism of molecule transcytosis inside the cells contains various pathways including caveolae-dependent endocytosis, clathrin-coated vesicle-mediated endocytosis and macropinocytosis. To further examine endocytic pathways for ZIKV entrance into barrier cells, we pretreated barrier cells with pharmacological inhibitors including Nystatin, chlorpromazine (CPZ), and dimethyl amiloride (DMA), which specifically inhibit caveolae-dependent endocytosis, clathrin-dependent endocytosis, and micropinocytosis, respectively. Colchicine, an inhibitor to prevent microtubule polymerization was used to disrupt intracellular trafficking. The concentration of inhibitors used in the current study did not cause cytotoxicity or increase cellular permeability in both JEG-3 and hCMEC/D3 cells (Figures 7A,B). Compared to the control group, the treatment of the three pharmacological inhibitors of endocytosis decreased the intensity of atto647N signals in the basal chamber of a Transwell plate (Figure 7C), suggesting that endocytosis might be essential for the ZIKV to cross the monolayer of barrier cells. In addition, the treatment of colchicine also reduced atto647N intensity in the basal chamber (Figure 7C), which indicated the requirement of microtubule polymerization for intracellular trafficking of the ZIKV. To further quantify the effect of inhibitors on the ZIKV crossing barrier cells, the titers of ZIKV isolated from the basal chamber 3 h post-infection were determined by a plaque assay. The results depicted that all four inhibitors reduced viral titers in the basal chamber, compared to that in the control group (Figure 7D). Taken together, these results suggested that transcytosis may be an important pathway for the ZIKV to cross the placental barrier and the BBB.

## Discussion

**FIGURE 8:**
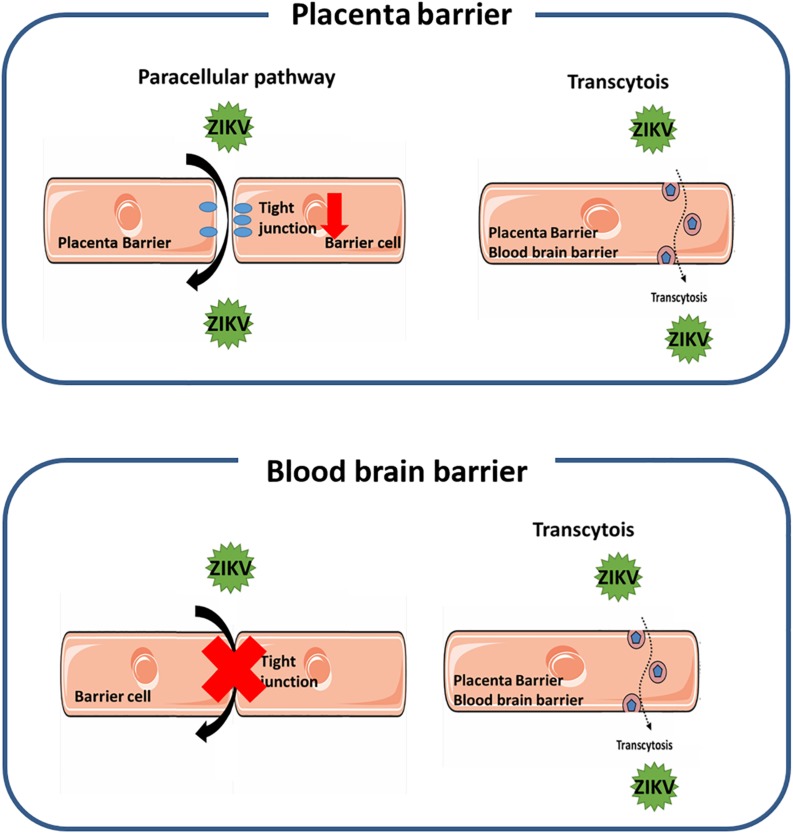
A schematic diagram depicts the mechanism of the ZIKV crossing human physiological barriers. In the placenta barrier, ZIKV infection can lead to disruption of tight junction that results in the ZIKV crossing through the leakage of tight junction. In addition, the ZIKV can also transport from the apical side to the basolateral side through the transcytosis pathway (top panel). In the blood-brain barrier, the ZIKV can transport from the apical side to the basolateral side through the transcytosis pathway, but does not alter the permeability of the barrier (bottom panel).

In this study we investigated the pathways for the ZIKV crossing the endothelial cell monolayer of both the placenta barrier and the BBB. Using fluorescence-tagged dextran as an indicator, our results revealed that ZIKV infection enhanced cellular permeability in JEG-3 cells but not in hCMEC/D3 cells. Moreover, in JEG-3 cells, ZIKV infection reduces the amount of tight junction proteins, ZO-1 and occludin, through the proteasomal degradation pathway, resulting in disruption of tight junction. In contrast, using fluorescence-labeled ZIKV particles and pharmacological inhibitors of endocytosis, we demonstrated transcytosis as an additional pathway for the ZIKV crossing the monolayer of JEG-3 and hCMEC/D3. As summarized in Figure 8, our current study elucidated that the ZIKV uses a cell-type specific paracellular pathway to cross the placenta monolayer barrier by disrupting cellular tight junction. In addition, the ZIKV can also cross both the placenta barrier and the BBB by transcytosis (Figure 6). The present study provides new insights on cellular barrier penetration of ZIKV particles, which may facilitate the development of anti-ZIKV agents in the future.

Several lines of evidence showed the existence of ZIKV antigens in the chronic villi of a human placenta from a mother who gave birth to an infant with microcephaly (Calvet et al., 2016), and isolation of ZIKV RNA from placental tissue of mice infected with the ZIKV (Caine et al., 2018), suggesting that the ZIKV may penetrate the placental barrier to infect the infant brain. Recent studies reported that the ZIKV is able to infect and replicate in Hofbauer cells that are primary human placental macrophages and in cytotrophoblasts, suggesting a route of intrauterine transmission that the ZIKV crosses the fetal compartment by directly infecting the placental cells (Quicke et al., 2016). In this study, we revealed that the ZIKV could cross the placenta barrier with both paracellular and transcytosis (Figures 2, 5–7). Normally, microorganisms spreading through epithelial tissues are blocked by tight junctions and adherent junctions present on apical and basolateral surfaces, respectively (Mateo et al., 2015). However, our data revealed that ZIKV infection causes leakage of the placental barrier by disrupting the integrity of the tight junction of the barrier cells (Figures 2, 3). Further investigation elucidated that the breakdown of the tight junction in the placental barrier cells is due to a decrease in the amounts of ZO-1 and occludin, two essential tight junction proteins, through the proteasomal degradation pathway (Figure 4). In contrast to the direct infection pathway (Quicke et al., 2016), our results proposed a paracellular pathway for the ZIKV crossing the placental barrier by disrupting the cellular tight junction of the barrier cells through degradation of ZO-1 and occludin. The activation mechanism of the proteasomal degradation pathway by ZIKV infection remains to be further investigated.

Using a SVT approach, we provided evidence to elucidate that the ZIKV can cross the *in vitro* placenta model through transcytosis (Figure 6A). Maternal-fetal transmission of a number of viruses by transcytosis in the placenta has been proposed previously (Bhat and Anderson, 2007). Given that transport of maternal IgG across the placenta is minimal during the first trimester and rises dramatically between 22 and 26 weeks of gestation (Simister and Story, 1997; Palmeira et al., 2012), Matthew al. showed that DENV cross-reactive mAbs bound to the ZIKV undergo FcRn-mediated transcytosis across the placenta to productively infect human placental macrophages (Zimmerman et al., 2018). In the current study, the ZIKV crossing the placenta barrier cells was directly visualized by using Atto647N-ZIKV particles (Figure 6A). These results provide strong evidence demonstrating the straight passing of viral particles across the placental barrier rather than a release of newly produced viral particles after replication. Moreover, the action of ZIKV transcytosis could be inhibited by treatments with endocytosis inhibitors and colchicine (Figure 7). Taken together, we demonstrated that both the paracellular pathway and the transcytosis pathway are utilized by ZIKV to cross the placenta barrier (Figure 8, top panel). Further studies will be needed in order to illustrate how ZIKV particles select a pathway to cross the placenta barrier and whether they require a specific receptor for ZIKV to interact with.

In addition to the placenta barrier, the BBB is the other important barrier that protect the fetal brain development during pregnancy (Goasdoue et al., 2017). A number of neurotropic viruses enter the CNS by using various pathways including direct transport from peripheral nerves, transinfection and transcytosis (Mustafa et al., 2019). In the current study, the ZIKV can cross brain endothelial cells and release of infectious virus particles, without an increase of endothelial monolayer permeability and no significant cytotoxicity *in vitro* (Figures 1, 2, 7). This is in agreement with previous studies showing the ZIKV crosses the BBB monolayer without the BBB barrier disruption (Papa et al., 2017; Alimonti et al., 2018). However, our studies showed that there were infectious atto647N-labeled virus particles that crossed the monolayer of BBB barrier cells in the presence of the treatment of endocytic inhibitors (Figures 7C,D), suggesting that we still cannot exclude the possibility that some viral particles selectively modulate tight junctions and cross the bottom chamber via paracellular diapedesis without overtly disrupting the BBB permeability. Furthermore, although *in vivo* experiment models proposed that the ZIKV crosses the BBB with no severe disruption, barrier breakdown was detected at a later post-infection time (Papa et al., 2017). Because ZIKV infection may recruit leukocyte to the brain and induce neuron lesion and death (Jurado et al., 2018; Mustafa et al., 2019), it is possible that later BBB disruption may be triggered by inflammatory response rather than ZIKV-induced proteasomal degradation.

Understanding the pathways for ZIKV passing through physiological barriers, the placental barrier and the BBB, furthers our understanding of the pathophysiology of the ZIKV and provides a basis for developing anti-ZIKV drugs in a relevant cell type. Given that the activation of the proteasome degradation pathway to disrupt tight junction protein participates in a paracellular pathway for the maternal-fetal transmission of the ZIKV by disrupting tight junction proteins (Figures 2–4), it may lead to a new anti-ZIKV approach to maintain the integrity of tight junction and inhibit the process of viral extravasation in the placental by blocking degradation of the tight junction protein. In addition, our findings offer evidence that transcytosis may be a common strategy for the ZIKV to cross both the placental barrier and the BBB (Figures 5–7). Since transcytosis is a critical pathway to transport macromolecules intracellularly through the vesicular system (Tuma and Hubbard, 2003), it may not be a good druggable target to treat ZIKV infection. Therefore, further studies to reveal whether there is a specific interaction of the ZIKV with the transcytosis machine will facilitate the development of new anti-ZIKV agents.

## Data Availability Statement

The datasets generated for this study are available on request to the corresponding author.

## Author Contributions

C-FC, L-WC, C-CJ, and Y-HP designed the research, analyzed the data, and wrote the manuscript. C-FC, L-WC, and I-CL performed the research. C-CJ, YS, and Y-LL provided the critical reagents and assisted virus study.

## Conflict of Interest

The authors declare that the research was conducted in the absence of any commercial or financial relationships that could be construed as a potential conflict of interest.

## References

[B1] Alimonti J. B. Ribecco-Lutkiewicz M. Sodja C. Jezierski A. Stanimirovic D. B. Liu Q. (2018). Zika virus crosses an in vitro human blood brain barrier model. *Fluids Barriers CNS* 15:15. 10.1186/s12987-018-0100-y 29759080 PMC5952854

[B2] Al-Obaidi M. M. J. Bahadoran A. Har L. S. Mui W. S. Rajarajeswaran J. Zandi K. (2017). Japanese encephalitis virus disrupts blood-brain barrier and modulates apoptosis proteins in THBMEC cells. *Virus Res.* 233 17–28. 10.1016/j.virusres.2017.02.012 28279803

[B3] Ayloo S. Gu C. (2019). Transcytosis at the blood-brain barrier. *Curr. Opin. Neurobiol.* 57 32–38. 10.1016/0006-8993(87)90236-8 30708291 PMC6629499

[B4] Bhat P. Anderson D. A. (2007). Hepatitis B virus translocates across a trophoblastic barrier. *J. Virol.* 81 7200–7207. 10.1128/jvi.02371-06 17442714 PMC1933314

[B5] Brasil P. Sequeira P. C. Freitas A. D. Zogbi H. E. Calvet G. A. De Souza R. V. (2016). Guillain-Barre syndrome associated with Zika virus infection. *Lancet* 387:1482. 10.1016/S0140-6736(16)30058-7 27115821

[B6] Burton G. J. Fowden A. L. (2015). The placenta: a multifaceted, transient organ. *Philos. Trans. R. Soc. Lond. B Biol. Sci.* 370:20140066. 10.1098/rstb.2014.0066 25602070 PMC4305167

[B7] Caine E. A. Jagger B. W. Diamond M. S. (2018). Animal models of zika virus infection during pregnancy. *Viruses* 10:E598. 10.3390/v10110598 PMC6266710 30384472

[B8] Calvet G. Aguiar R. S. Melo A. S. O. Sampaio S. A. De Filippis I. Fabri A. (2016). Detection and sequencing of Zika virus from amniotic fluid of fetuses with microcephaly in Brazil: a case study. *Lancet Infect. Dis.* 16 653–660. 10.1016/S1473-3099(16)00095-5 26897108

[B9] Chu L. W. Huang Y. L. Lee J. H. Huang L. Y. Chen W. J. Lin Y. H. (2014). Single-virus tracking approach to reveal the interaction of Dengue virus with autophagy during the early stage of infection. *J. Biomed. Opt.* 19:011018. 10.1117/1.JBO.19.1.011018 24192777

[B10] Chu L. W. Yang C. J. Peng K. J. Chen P. L. Wang S. J. Ping Y. H. (2019). TIM-1 as a signal receptor triggers dengue virus-induced autophagy. *Int. J. Mol. Sci.* 20:4893. 10.3390/ijms20194893 31581681 PMC6801812

[B11] Cordeiro M. T. Pena L. J. Brito C. A. Gil L. H. Marques E. T. (2016). Positive IgM for Zika virus in the cerebrospinal fluid of 30 neonates with microcephaly in Brazil. *Lancet* 387 1811–1812. 10.1016/s0140-6736(16)30253-7 27103126

[B12] Coyne C. B. Lazear H. M. (2016). Zika virus - reigniting the TORCH. *Nat. Rev. Microbiol.* 14 707–715. 10.1038/nrmicro.2016.125 27573577

[B13] Daneman R. Prat A. (2015). The blood-brain barrier. *Cold Spring Harb. Perspect. Biol.* 7:a020412. 10.1101/cshperspect.a020412 25561720 PMC4292164

[B14] Daniels B. P. Klein R. S. (2015). Viral sensing at the blood-brain barrier: new roles for innate immunity at the CNS vasculature. *Clin. Pharmacol. Ther.* 97 372–379. 10.1002/cpt.75 25670037

[B15] Delorme-Axford E. Sadovsky Y. Coyne C. B. (2014). The Placenta as a barrier to viral infections. *Annu. Rev. Virol.* 1 133–146. 10.1146/annurev-virology-031413-085524 26958718

[B16] Dick G. W. Kitchen S. F. Haddow A. J. (1952). Zika virus. i. isolations and serological specificity. *Trans. R. Soc. Trop. Med. Hyg.* 46 509–520. 10.1016/0035-9203(52)90042-4 12995440

[B17] Dos Santos T. Rodriguez A. Almiron M. Sanhueza A. Ramon P. De Oliveira W. K. (2016). Zika virus and the guillain-barre syndrome - case series from seven countries. *N. Engl. J. Med.* 375 1598–1601. 27579558 10.1056/NEJMc1609015

[B18] Fitzgerald B. Boyle C. Honein M. A. (2018). Birth defects potentially related to zika virus infection during pregnancy in the united states. *JAMA* 319 1195–1196. 29372233 10.1001/jama.2018.0126 PMC5988243

[B19] Goasdoue K. Miller S. M. Colditz P. B. Bjorkman S. T. (2017). Review: the blood-brain barrier; protecting the developing fetal brain. *Placenta* 54 111–116. 10.1016/j.placenta.2016.12.005 27939102

[B20] Gude N. M. Roberts C. T. Kalionis B. King R. G. (2004). Growth and function of the normal human placenta. *Thromb. Res.* 114 397–407. 10.1016/j.thromres.2004.06.038 15507270

[B21] Hartsock A. Nelson W. J. (2008). Adherens and tight junctions: structure, function and connections to the actin cytoskeleton. *Biochim. Biophys. Acta* 1778 660–669. 10.1016/j.bbamem.2007.07.012 17854762 PMC2682436

[B22] Hoen B. Schaub B. Funk A. L. Ardillon V. Boullard M. Cabie A. (2018). Pregnancy outcomes after ZIKV infection in french territories in the americas. *N. Engl. J. Med.* 378 985–994. 10.1056/NEJMoa1709481 29539287

[B23] Jurado K. A. Yockey L. J. Wong P. W. Lee S. Huttner A. J. Iwasaki A. (2018). Antiviral CD8 T cells induce Zika-virus-associated paralysis in mice. *Nat. Microbiol.* 3 141–147. 10.1038/s41564-017-0060-z 29158604 PMC5780207

[B24] Leibrand C. R. Paris J. J. Ghandour M. S. Knapp P. E. Kim W.-K. Hauser K. F. (2017). HIV-1 Tat disrupts blood-brain barrier integrity and increases phagocytic perivascular macrophages and microglia in the dorsal striatum of transgenic mice. *Neurosci. Lett.* 640 136–143. 10.1016/j.neulet.2016.12.073 28057474 PMC5401762

[B25] Liou M. L. Hsu C. Y. (1998). Japanese encephalitis virus is transported across the cerebral blood vessels by endocytosis in mouse brain. *Cell Tissue Res.* 293 389–394. 9716728 10.1007/s004410051130

[B26] Mateo M. Generous A. Sinn P. L. Cattaneo R. (2015). Connections matter–how viruses use cell-cell adhesion components. *J. Cell Sci.* 128 431–439. 10.1242/jcs.159400 26046138 PMC4311127

[B27] Mittal R. Nguyen D. Debs L. H. Patel A. P. Liu G. Jhaveri V. M. (2017). Zika virus: an emerging global health threat. *Front. Cell Infect. Microbiol.* 7:486. 10.3389/fcimb.2017.00486 29276699 PMC5727043

[B28] Mlakar J. Korva M. Tul N. Popovic M. Poljsak-Prijatelj M. Mraz J. (2016). Zika virus associated with microcephaly. *N. Engl. J. Med.* 374 951–958. 10.1056/NEJMoa1600651 26862926

[B29] Morad G. Carman C. V. Hagedorn E. J. Perlin J. R. Zon L. I. Mustafaoglu N. (2019). Tumor-derived extracellular vesicles breach the intact blood-brain barrier via transcytosis. *ACS Nano* 13 13853–13865. 10.1021/acsnano.9b04397 31479239 PMC7169949

[B30] Mustafa Y. M. Meuren L. M. Coelho S. V. A. De Arruda L. B. (2019). Pathways exploited by flaviviruses to counteract the blood-brain barrier and invade the central nervous system. *Front. Microbiol.* 10:525. 10.3389/fmicb.2019.00525 30984122 PMC6447710

[B31] Palmeira P. Quinello C. Silveira-Lessa A. L. Zago C. A. Carneiro-Sampaio M. (2012). IgG placental transfer in healthy and pathological pregnancies. *Clin. Dev. Immunol.* 2012:985646. 10.1155/2012/985646 22235228 PMC3251916

[B32] Papa M. P. Meuren L. M. Coelho S. V. A. Lucas C. G. O. Mustafa Y. M. Lemos Matassoli F. (2017). Zika virus infects, activates, and crosses brain microvascular endothelial cells, without barrier disruption. *Front. Microbiol.* 8:2557. 10.3389/fmicb.2017.02557 29312238 PMC5743735

[B33] Petersen L. R. Jamieson D. J. Powers A. M. Honein M. A. (2016). Zika virus. *N. Engl. J. Med.* 374 1552–1563. 27028561 10.1056/NEJMra1602113

[B34] Quicke K. M. Bowen J. R. Johnson E. L. Mcdonald C. E. Ma H. O’Neal J. T. (2016). Zika virus infects human placental macrophages. *Cell Host Microbe* 20 83–90. 10.1016/j.chom.2016.05.015 27247001 PMC5166429

[B35] Roe K. Kumar M. Lum S. Orillo B. Nerurkar V. R. Verma S. (2012). West nile virus-induced disruption of the blood-brain barrier in mice is characterized by the degradation of the junctional complex proteins and increase in multiple matrix metalloproteinases. *J. Gen. Virol.* 93 1193–1203. 10.1099/vir.0.040899-0 22398316 PMC3755517

[B36] Roe K. Orillo B. Verma S. (2014). West nile virus-induced cell adhesion molecules on human brain microvascular endothelial cells regulate leukocyte adhesion and modulate permeability of the in vitro blood-brain barrier model. *PLoS One* 9:e102598. 10.1371/journal.pone.0102598 25036379 PMC4103843

[B37] Rothbauer M. Patel N. Gondola H. Siwetz M. Huppertz B. Ertl P. (2017). A comparative study of five physiological key parameters between four different human trophoblast-derived cell lines. *Sci. Rep.* 7:5892. 10.1038/s41598-017-06364-z 28724925 PMC5517571

[B38] Sevvana M. Long F. Miller A. S. Klose T. Buda G. Sun L. (2018). Refinement and analysis of the mature zika virus cryo-em structure at 3.1 a resolution. *Structure* 26 1169–1177.e3. 10.1016/j.str.2018.05.006 29958768 PMC6125166

[B39] Simister N. E. Story C. M. (1997). Human placental Fc receptors and the transmission of antibodies from mother to fetus. *J. Reprod. Immunol.* 37 1–23. 10.1016/s0165-0378(97)00068-5 9501287

[B40] Sirohi D. Chen Z. Sun L. Klose T. Pierson T. C. Rossmann M. G. (2016). The 3.8 A resolution cryo-EM structure of Zika virus. *Science* 352 467–470. 10.1126/science.aaf5316 27033547 PMC4845755

[B41] Soares C. N. Brasil P. Carrera R. M. Sequeira P. De Filippis A. B. Borges V. A. (2016). Fatal encephalitis associated with Zika virus infection in an adult. *J. Clin. Virol.* 83 63–65. 10.1016/j.jcv.2016.08.297 27598870

[B42] Stamatovic S. M. Keep R. F. Andjelkovic A. V. (2008). Brain endothelial cell-cell junctions: how to “open” the blood brain barrier. *Curr. Neuropharmacol.* 6 179–192. 10.2174/157015908785777210 19506719 PMC2687937

[B43] Tuma P. Hubbard A. L. (2003). Transcytosis: crossing cellular barriers. *Physiol. Rev.* 83 871–932. 10.1152/physrev.00001.2003 12843411

[B44] Vu K. Weksler B. Romero I. Couraud P. O. Gelli A. (2009). Immortalized human brain endothelial cell line HCMEC/D3 as a model of the blood-brain barrier facilitates in vitro studies of central nervous system infection by *Cryptococcus neoformans*. *Eukaryot. Cell* 8 1803–1807. 10.1128/EC.00240-09 19767445 PMC2772405

[B45] Zimmerman M. G. Quicke K. M. O’neal J. T. Arora N. Machiah D. Priyamvada L. (2018). Cross-reactive dengue virus antibodies augment zika virus infection of human placental macrophages. *Cell Host Microbe* 24 731–742.e6. 10.1016/j.chom.2018.10.008 30439342 PMC6394860

